# Neurophysiological predictors of full clinical consciousness recovery in patients with DoC: a retrospective evaluation of the DoC-NP Score

**DOI:** 10.3389/fneur.2026.1795913

**Published:** 2026-06-19

**Authors:** Valerio Sveva, Ilaria Sermasi, Valentina Colombo, Francesca Cesira Cava, Bledi Shehaj, Alice Rita Portillo, Chiara Minardi, Matteo Bologna, Paola Rucci, Pamela Salucci

**Affiliations:** 1 Severe Acquired Brain Injury Unit, Montecatone Rehabilitation Institute S.p.A., Imola, Italy; 2 Department of Human Neurosciences, Sapienza University of Rome, Rome, Italy; 3 Clinical Neurophysiology Inpatient Unit, Montecatone Rehabilitation Institute S.p.A., Imola, Italy; 4 Istituto di Ricovero e Cura a Carattere Scientifico (IRCSS) Neuromed, Pozzilli, Italy; 5 Department of Biomedical and Neuromotor Sciences (DIBINEM) Physiology Unit, University of Bologna, Bologna, Italy

**Keywords:** DoC, EEG, ERPs, MCS, prognostication, sABI, SEPs, UWS/VS

## Abstract

**Background:**

Prognostication in prolonged disorders of consciousness (pDoC) remains challenging, particularly when estimating recovery of a clinically observable conscious state. Multimodal neurophysiological markers may provide complementary prognostic information beyond behavioral assessment alone.

**Objective:**

To develop and retrospectively evaluate a composite neurophysiological score (DoC-NP Score) for predicting emergence from the minimally conscious state (eMCS) during post-acute neurorehabilitation.

**Methods:**

This retrospective cohort study included 76 patients with pDoC following severe acquired brain injury (sABI). Clinical assessment using the Coma Recovery Scale–Revised (CRS-R) was performed at admission (T0) and discharge (T1). Neurophysiological evaluation at T0 included EEG, upper-limbs somatosensory evoked potentials (SEPs), and auditory event-related potentials (ERPs). The DoC-NP Score integrates standard EEG features (continuity, amplitude, dominant frequency and reactivity), N20 SEPs, and P300 ERPs into a composite ordinal score. Associations between the DoC-NP Score, eMCS recovery, and functional outcomes (LCF, DRS) were analyzed using regression models and ROC analysis.

**Results:**

Higher DoC-NP scores at T0 were associated with greater likelihood of eMCS at T1 (*OR* = 1.768 per point; AUC = 0.768, optimal cut-off = 6) and independently predicted better functional (LCF, *p* < 0.005) and disability (DRS, *p* < 0.025) outcomes. eMCS was achieved at T1 by 35 patients (46%), independent of injury etiology (*p* > 0.05). EEG AFR (*r* = 0.854), EEG continuity (*r* = 0.668) and SEPs (*r* = 0.673) contributed most to the prognostic value of the composite score, while ERPs showed moderate (*r* = 0.489). Moreover, the DoC-NP score correlates moderately with the change in CRS-R total score (*r* = 0.434, *p* < 0.001), auditory function (*r* = 0.445, *p* < 0.001), motor function (*r* = 0.435, *p* < 0.001), motor oral/verbal function (*r* = 0.334, *p* = 0.003), communication (*r* = 0.315, *p* = 0.003); poorly with visual function (*r* = 0.27, *p* = 0.016) and no correlation with vigilance (*p* = 0.168).

**Conclusions:**

The DoC-NP Score is a feasible multiparametric neurophysiological tool that may support prognostic stratification in pDoC patients as a complementary component to multimodal assessment, although its modest incremental predictive value requires prospective validation.

## 1 Introduction

Disorders of Consciousness (DoC) comprise a heterogeneous group of clinical conditions characterized by impaired arousal and/or awareness resulting from severe acquired brain injury (sABI) (1). The most common etiologies include post-hypoxic brain injury following cardiac arrest, traumatic brain injury (TBI), intracerebral hemorrhage, and ischemic stroke (2, 3). DoC frequently led to long-term or permanent disability, posing major clinical, ethical, and socioeconomic challenges (4). When altered consciousness persists for more than 28 days, the condition is classified as prolonged DoC (pDoC), which may last for months or even throughout the patient's lifetime (5).

Clinically, the levels of consciousness are categorized into discrete diagnostic entities. Coma is defined by the absence of both arousal and awareness; the vegetative state (VS), also referred to as unresponsive wakefulness syndrome (UWS), is characterized by preserved arousal without behavioral evidence of awareness; the minimally conscious state (MCS) is marked by inconsistent but reproducible signs of awareness; and emergence from MCS (eMCS) is identified by the recovery of functional communication or functional object use (1, 6). This diagnostic framework relies on standardized neurobehavioral assessment tools, particularly the Coma Recovery Scale–Revised (CRS-R), which identifies subtle behavioral signs of consciousness that may be missed during routine clinical examination (7). In the context of DoC, it is important to distinguish between consciousness as a brain state and consciousness as a broader mental ability. Clinical assessment primarily targets the recovery of a conscious brain state, operationally defined by the restoration of consistent behavioral responsiveness to environmental stimuli (8). This operational definition differs from the broader concept of full human consciousness, which refers to a complex mental capacity encompassing awareness, intentionality, and higher-order cognitive functions that have evolved across species (9).

Following diagnostic classification made in the acute phase in the Intensive Care Units (ICUs), a key clinical task is neuroprognostication during the sub-acute phase in the Intensive Rehabilitation Unit (IRUs), defined as estimating the likelihood and trajectory of consciousness recovery (10). Recovery trajectories are influenced by multiple contextual and clinical factors, including age, etiology of injury, pre-morbid functional status, comorbidities, and medical complications (2, 10). Moreover, prognostic evaluation increasingly incorporates markers of functional and structural brain integrity (11). However, no single prognostic marker demonstrates sufficient accuracy when used in isolation, highlighting the need for multimodal approaches that combine complementary sources of information (12–14).

After clinical stabilization, patients with DoC are typically transferred from the ICU to an IRU, in order to maximize functional outcomes and to regain autonomies. In this setting, clinicians are required to estimate expected outcomes in order to inform families and to design individualized rehabilitation pathways that account for clinical benefit, ethical considerations, and healthcare resource allocation (15). Early identification of patients more likely to recover consciousness is particularly relevant for rehabilitation planning. During the rehabilitation phase, functional and disability outcomes are commonly monitored using the Levels of Cognitive Functioning (LCF) scale and the Disability Rating Scale (DRS), in conjunction with repeated CRS-R assessments (16, 17).

Prognostication is particularly challenging in patients who remain unresponsive. Behavioral assessment, although considered the clinical reference standard, may be limited by motor impairments, aphasia, sensory deficits, fluctuating arousal, intercurrent medical complications, and subjective interpretation of ambiguous behaviors (18–20). For these reasons, recent clinical guidelines recommend a multimodal assessment of consciousness, in which behavioral evaluations are complemented by instrumental investigations (6, 21).

Recent studies have empashized the importance of multimodal prognostic approaches in DoC, combining clinical examination, electrophysiology, and neuroimaging—including paradigms capable of detecting Covert Awareness, also known as Covert Consciousness, Cognitive Motor Dissociation (CMD), functional Locked-In Syndrome (fLIS) or Non-Behavioral MCS (MCS^*^) (13, 22–24). However, the availability of such comprehensive assessments varies across different clinical settings and often depend on local resources, infrastructure, and specialized expertise (19).

A broad range of neurophysiological and neuroimaging markers has been proposed for prognostication in DoC, including Electroencephalographic (EEG) reactivity to passive stimulation (25), entropy-based measures (26), dominant background frequency (27), somatosensory evoked potentials (SEPs) (28–30), long-latency auditory event-related potentials (ERPs, such as Mismatch Negativity–MMN and P300) (31), non-rapid eye movement (N-REM) features (such as sleep spindles and K-complexes) (32), perturbational complexity index (PCI) using transcranial magnetic stimulation (TMS) coupled with EEG (TMS-EEG) (33), and neuroimaging techniques such as functional magnetic resonance imaging (fMRI), and positron emission tomography/single-photon emission computed tomography (PET/SPECT) (34, 35). While these approaches provide valuable and complementary insights into brain function, their prognostic performance is heterogeneous, and many measures show variable reliability when considered in isolation, with substantial inter-study variability (36). Moreover, most studies have relied on univariate approaches, potentially underestimating the contribution of individual markers when interpreted within a broader multimodal context, thus supporting the need for integrated prognostic strategies combining clinical, neurophysiological, and neuroimaging data (37).

In this context, the present study aimed to develop a feasible multivariate composite neurophysiological score integrating EEG features, the assessment of thalamo-cortical pathway integrity through N20 SEPs, and cognitive ERPs such as the P300 component, to provide a structured bedside tool that may complement existing clinical and neuroimaging-based prognostic evaluations during the rehabilitation phase (28, 38, 39). Unlike many previous observational studies focused their endpoint as “improvement of consciousness”, this study specifically targeted “emergence from the minimally conscious state” (eMCS) as a clinically meaningful endpoint reflecting recovery of a full clinical observable conscious state (40).

Accordingly, aim of this retrospective study was to develop and validate a composite neurophysiological score, the DoC-NP Score, to support prognostic assessment of recovery of a clinically observable state of consciousness, operationally defined as eMCS according to CRS-R diagnostic criteria. The DoC-NP Score integrates standard 30-min bedside electroencephalographic (EEG) features with the presence or absence of N20 somatosensory evoked potentials (SEPs) and the presence or absence of the P300 event-related potential (ERP). The primary aim was to evaluate its association with eMCS, as assessed by the CRS-R. The secondary aim was to examine the relationships with functional independence and disability outcomes, measured by LCF and DRS scales.

## 2 Materials and methods

### 2.1 Study design and setting

This retrospective cohort study was conducted over an approximately 10-year period, from January 1, 2015, to June 30, 2025. The study included patients with DoC admitted to a tertiary referral specialized rehabilitation hospital, the Montecatone Rehabilitation Institute S.p.A. (Imola, Italy). All patients were hospitalized in the inpatient post-acute severe acquired brain injury (sABI) unit and were evaluated in collaboration with the institute's inpatient clinical neurophysiology unit.

### 2.2 Study population

Eligible participants met the following inclusion criteria: (1) age ≥ 18 years; (2) clinical diagnosis of VS/UWS or MCS, established using the CRS-R at both admission and discharge; (3) LCF score ≤ 3 at admission; (4) DoC resulting from sABI of any etiology (traumatic, vascular, anoxic, infectious, neoplastic, or mixed); (5s) time from acute brain injury between 28 days (meeting criteria for prolonged DoC) and 6 months; and (6) availability of at least two complete sets of clinical (CRS-R, DRS, and LCF) performed at admission (T0) and prior to discharge (T1); (7) a full neurophysiological (EEG, SEPs, ERPs) evaluations at admission (T0).

Exclusion criteria included: (1) age < 18 years at the time of injury or admission; (2) pre-existing neurodegenerative disorders (e.g., Alzheimer's disease, Parkinson's disease) or non-acquired causes of altered consciousness (e.g., congenital, metabolic, or toxic encephalopathies); (3) history of epilepsy; (4) pre-existing severe psychiatric disorders (e.g., major depressive disorder, schizophrenia, bipolar disorder). Patients who did not complete the full neurophysiological assessment battery (EEG, SEPs, and ERPs) at admission (T0) were excluded. In addition, patients who developed unstable medical conditions that could interfere with reliable assessment of consciousness—such as hemodynamic instability, severe respiratory failure, status epilepticus or uncontrolled seizures, or central nervous system infection—were excluded from the analysis.

### 2.3 The DoC-NP Score

The DoC-NP Score is a composite neurophysiological score integrating four EEG features with somatosensory and cognitive evoked potentials. Specifically, the score combines EEG continuity, amplitude, dominant frequency and reactivity, with the presence of upper-limb N20 SEPs and the cognitive P300 ERP. The DoC-NP Score is an ordinal scale with increasing values corresponding to more favorable neurophysiological profiles, ranging from a minimum score of 1 (worst performance) to a maximum score of 11 (best performance).

From EEG recordings, the following parameters were evaluated:

- Continuity was classified according to the American Clinical Neurophysiology Society (ACNS) Critical Care EEG terminology as described by Hirsch et al. (41). Within the scoring system, a continuous pattern was assigned 3 points, nearly-continuous 2 points, and discontinuous 1 point. Burst-suppression and suppression patterns were combined and considered the least favorable prognostic category, receiving 0 points.
- EEG background characteristics were derived from the previously described Amplitude–Frequency–Reactivity (AFR) score proposed by Bagnato et al. (25). Amplitude was considered normal when ≥ 20 μV (1 point) and reduced when < 20 μV in the majority of channels (0 points). Dominant frequency was defined as the most prevalent frequency band across channels, with alpha activity (8–12 Hz) scoring 2 points, theta activity (4–7 Hz) scoring 1 point, and delta activity (< 4 Hz) scoring 0 points. Reactivity was considered present when a reproducible change in EEG amplitude or frequency occurred following at least one auditory or nociceptive stimulus, yielding 1 point; absence of reactivity was scored as 0.

SEPs were evaluated by assessing the presence of the cortical N20 response following median nerve stimulation of the upper limbs, which represents the SEP component with established prognostic value. The presence of bilateral N20 responses was assigned 2 points; unilateral presence or bilateral responses with reduced amplitude were assigned 1 point. Given the well-established evidence that the bilateral absence of the N20 SEP is a strong predictor of poor outcome in post-hypoxic cardiac arrest, but not consistently in traumatic brain injury, the absence of bilateral N20 responses was not explicitly scored as a zero-point condition in the present scoring system (29, 30, 42).

ERPs were assessed using an auditory oddball paradigm. The presence of both the auditory N100 and P300 components was assigned 2 points. The presence of N100 in the absence of P300 was assigned 1 point, whereas the absence of both components was assigned 0 points. ERPs were acquired following the assessment of short-latency brainstem auditory evoked potentials (BAEPs), which was performed to verify the functional integrity of the auditory sensory pathway.

All neurophysiological parameters and the corresponding scores assigned to each category are summarized in Table 1. The total score is the sum of the sub-scores of neurophysiological parameters.

**Table 1:** Overview of the DoC-NP score and its parameters.

DoC-NP SCORE	Score
EEG–continuity	
Continuous	3
Nearly continuous	2
Discontinuous	1
Burst-suppression/suppression	0
EEG–AFR	
Amplitude	
Normal (≥20 uV)	1
Reduced (< 20 uV)	0
Dominant frequency	
Alpha (8–12 Hz)	2
Theta (4–7 Hz)	1
Delta (< 4 Hz)	0
Reactivity	
Present	1
Absent	0
SEPs–N20	
Present bilaterally	2
Present monolaterally/bilaterally with low-voltage	1
ERPs–N100 and P300	
N100 and P300 present	2
N100 present, P300 absent	1
N100 and P300 absent	0

The total score is the sum of the parameter scores and ranges from 1 to 11. EEG, electroencelography; SEPs, somatosensory evoked potentials; ERPs, evoked-related potentials.

#### 2.3.1 Electroencephalogram (EEG)

Standard EEG recordings of 30-min duration were acquired using a digital workstation (Be Plus Pro, EBNeuro S.p.A., Florence, Italy) and a pre-wired EEG cap with 21 electrodes (19 active electrodes, one ground, and one reference), positioned according to the International 10–20 System (Fp1, Fp2, Fz, F3, F4, Cz, C3, C4, Pz, P3, P4, O1, O2, F7, F8, T3, T4, T5, and T6). Signals were sampled at a rate of 128 Hz. EEG recordings were obtained with patients' eyes closed and included three sequential conditions: (1) a 10-min resting-state segment; (2) 10 min of passive eyelid opening and closure; and (3) 10 min of sensory stimulation, consisting of auditory stimuli (verbal commands and hand clapping) and nociceptive stimuli (applied to the eyebrow or nail bed). An electrocardiogram (ECG) channel was simultaneously recorded to facilitate identification of cardiac-related artifacts. Long-term antiseizure medications were maintained throughout the recording period according to clinical indications. EEG signals were filtered using a high-pass filter with a time constant of 0.1–0.3 s and a low-pass filter with a cutoff frequency of 30–70 Hz, adjusted as needed for interpretative purposes. Recordings were displayed using a standard gain of 7 μV/mm and sensitivity settings ranging from 2 to 10 μV/mm. EEG patterns were classified and labeled in accordance with the revised 2021 ACNS Critical Care EEG terminology, as described by Hirsch et al. (41).

#### 2.3.2 Somatosensory evoked potentials (SEPs)

SEPs were recorded using a Medelec Synergy (Viatys Healthcare, USA). SEPs were recorded at peripheral, spinal, subcortical, and cortical levels using needle electrodes. The protocol used at Montecatone Rehabilitation Institute included 4 recording channels:

- Ch 1: contralateral cortical electrode referenced to ipsilateral cortex;
- Ch 2: ipsilateral cortical electrode referenced to contralateral Erb's point;
- Ch 3: spinal electrode at Cv6 referenced to contralateral Erb's point;
- Ch 4: ipsilateral Erb's point referenced to contralateral Erb's point.

Stimulation consisted of repetitive bipolar pulses applied to the median nerve at the wrist (3 Hz frequency, 0.2 ms pulse duration). When the sensory threshold could not be determined, stimulation intensity was increased until a visible twitch of the abductor pollicis brevis (APB) muscle was observed. In cases in which no muscular twitch could be elicited (e.g., due to edema or polyneuropathy), stimulation intensity was increased to the maximum level allowed by the device.

#### 2.3.3 Event-related potentials (ERPs)

ERPs were recorded using the same digital workstation for recording EEG (Be Plus Pro, EBNeuro S.p.A., Florence, Italy), acquiring cortical activity from midline electrodes Fz, Cz, and Pz. An electrooculogram (EOG) channel was simultaneously recorded to enable offline identification and removal of ocular and blink-related artifacts. Auditory stimulation was delivered using a standard oddball paradigm. The stimulus sequence consisted of frequent non-target tones (480 stimuli, representing 80% of the total; 100 ms duration; 1 kHz frequency) randomly interspersed with infrequent target tones (120 stimuli, representing 20% of the total; 100 ms duration; 2 kHz frequency). Patients were instructed to attend to the target stimuli and, when possible, to silently count their occurrence. Offline signal processing was performed using NPX ERP Analysis software (EBNeuro S.p.A., Florence, Italy) to extract the auditory N100 and P300 components.

### 2.4 Clinical and neurophysiological data collection

Study data were retrieved from patients' digital medical records. At enrollment, demographic variables (age, sex) and clinical information, including etiology of brain injury and time since injury, were collected from medical records.

Within 1 week of study entry (T0), patients underwent repeated CRS-R assessments (at least five evaluations over 1 week) performed by trained and experienced examiners (V.C., F.C.C., B.S.) to confirm diagnostic classification and to identify the highest CRS-R subscale scores. The LCF and DRS were also recorded at T0.

Neurophysiological evaluations, including standard 30-min bedside EEG recordings, SEPs, and ERPs, were performed in different days at T0 by experienced neurophysiologist (EEG, SEPs, and ERPs: C.M., I.S, A.R.P.). Assessments were conducted as soon as possible after T0 due to logistical or organizational constraints.

Follow-up evaluations (T1) were performed at hospital discharge and included the same clinical scales (CRS-R, LCF, and DRS).

#### 2.4.1 Statistical analysis

The sample size calculation indicated that a minimum of 58 participants was required to perform multiple linear regression analyses with four predictors, assuming an 80% statistical power, a two-sided alpha level of 0.05, and an expected proportion of explained variance (*R*^2^) of 0.20 (G^*^Power v.3.1, HHU, Düsseldorf, Germany). Descriptive statistics, including mean and standard deviation or median and interquartile range were applied as appropriate. Between-group comparisons were performed using the chi-square test for categorical variables, the independent-samples t-test for normally distributed continuous variables, and Mann–Whitney's *U*-test for non-normally distributed variables. Normality was tested using Shapiro-Wilk's test and the equality of variance using Brown-Forsyte test. To control for multiple comparisons, the Benjamini–Hochberg procedure was applied to adjust *p*-values.

Logistic and linear regression models were employed to examine the association of the DoC-NP Score with eMCS, measured by the CRS-R, and functional outcomes at discharge, using the LCF and DRS scales. Receiver operating characteristic (ROC) curve analysis was conducted to identify the optimal DoC-NP Score cut-off value discriminating patients who achieved full clinical recovery of consciousness and eMCS.

Spearman's rho heatmaps were used to visually represent the pattern and strength of correlations between the DoC-NP Score and its neurophysiological sub-items, and as well between total and individual sub-items of the CRS-R. Correlations below 0.30 can be considered weak, >0.30–0.50 moderate, >0.50 strong.

All statistical analyses were conducted using JASP, version 0.19.1.0 (University of Amsterdam, Copyright 2013–2024) and IBM SPSS, version 30.0, Copyright IBM Corporation, 1989–2024.

### 2.5 Ethical considerations

The study was approved by the local ethics committee (371–2025-OSS-AUSLIM - 25071), in accordance of the Declaration of Helsinki, and did not receive any funding. When applicable, informed consent was obtained from the legally authorized representative or court-appointed administrator. In cases where consent could not be obtained, data use was authorized by the Ethics Committee in accordance with national regulations for retrospective observational studies.

## 3 Results

**Figure 1:**
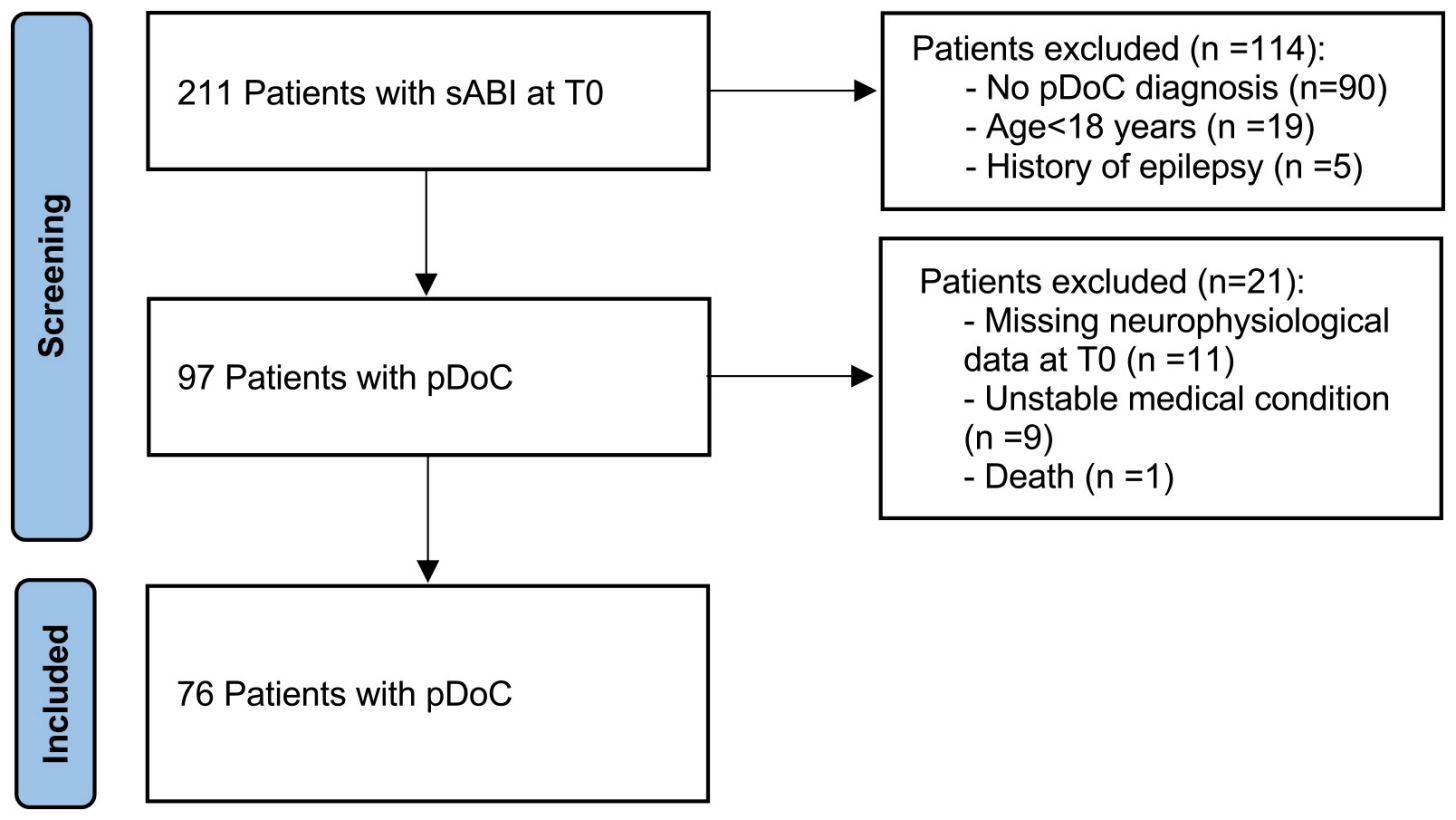
Study enrollment flowchart. sABI, severe acquired brain injury; pDoC, prolonged disorder of consciousness.

A total of 211 patients with sABI were admitted to the Montecatone Rehabilitation Institute's IRU from January 1, 2015 to June 30, 2025. After applying exclusion criteria, 97 patients fulfilled inclusion criteria at T0. Moreover, 21 additional patients were excluded because of missing neurophysiological data at T0 (*n* = 11), for unstable medical condition (*n* = 9), and for death (*n* = 1) during permanence in the IRU. Figure 1 shows the study enrollment flowchart.

Finally, the retrospective cohort included 76 patients [29 *F*_(38, 2%)_], with a mean age of 51.4 years (*SD* = 7.6, range 19–79). The etiology was: 38 (50%) traumatic and 38 (50%) non-traumatic (of which: 16 [21%] patients with post-hypoxia cardiac arrest and 22 [29%] patients with vascular stroke, where 3 [4%] of them were ischemic and 19 [25%] were hemorrhagic). The median distance from the sABI event was 81 days (IQR 60–115 days) from the evaluation at admission (T0), while the median length of stay at Montecatone Rehabilitation Institute was 200 days (IQR 160–278 days). At admission (T0), 44 patients (58%) were in VS/UWS and 32 of them (42%) in MCS.

Patients with MCS were significantly older (*p* < 0.05), they had a higher performance on the CRS-R parameters (*p* < 0.05), a higher DoC-NP Score (*p* < 0.05) and better EEG, SEP, ERP parameters (*p* < 0.05) compared to VS/UWS. On the contrary, no statistical difference was found between patients with traumatic and non-traumatic etiology (ischemic, vascular and post-anoxic), except for a higher mean age in the latter (*p* < 0.05).

Full clinical recovery of consciousness with eMCS was achieved at discharge (T1) by 35 patients (46%) and the association between recovery and etiology either categorized as traumatic, vascular hemorrhage, vascular ischaemia, anoxic (chi-square = 0.750, *p* = 0.861) or as traumatic/non-traumatic (chi-square = 0.213, *p* = 0.645) was not statistically significant. Instead, 22 patients (29%) remained in MCS, while 19 patients (25%) in VS/UWS.

Patients completed the neurophysiological battery in a median of 7 days (IQR 3–12) from admission to the IRU. The mean DoC-NP Score was 5.5 (*SD* = 2.2, range 1–10). The DoC-NP Score was positively correlated with age (Spearman's rho = 0.350, *p* = 0.002), uncorrelated with traumatic etiology (rho = −0.124, *p* = 0.287) and negatively correlated with the time (in days) from the lesion (Spearman's rho = 0.348, *p* = 0.002). Table 2 shows the demographic and clinical characteristics of the study cohort.

**Table 2:** Demographic, clinical and neurophysiological characteristics of the patients' cohort at admission (T0) by clinical diagnosis and etiology.

Patients' Characteristics	VS/UWS		MCS		Non-traumatic		Traumatic	
	Mean	SD/%	Mean	SD/%	Mean	SD/%	Mean	SD/%
Age	47.2	17.3	57.8^*^	15.5	58.5^*^	14.2	44.9	17.6
Sex								
F	13	29.5%	16	50,00%	19	50.0%	10	29.0%
M	31	70.5%	16	50,00%	19	50.0%	28	71.0%
Etiology								
Anoxia	11	25.0%	5	15,60%	16	42.1%	—	—
Vascular (Hemorrhage)	8	18.2%	11	34,40%	19	50,00%	—	—
Vascular (ischemia)	3	6.8%	—	—	3	7.9%	—	—
Trauma	22	50.0%	16	50,00%	—	—	38	100,00%
CRS-R								
Total	5.1	0.6	11.1^*^	0.7	7.7	0.9	7.5	0.8
Auditory function	1.0	0.5	2.0^*^	0.6	1.4	0.7	1.4	0.9
Visual function	0.8	0.9	2.5^*^	0.7	1.5	1.2	1.5	1.1
Motor function	1.2	0.7	2.5^*^	0.9	1.7	1.1	1.8	1.0
Oral/verbal function	0.8	0.6	1.5^*^	0.6	1.1	0.9	1.0	0.5
Communication	0.0	0.2	0.5^*^	0.5	0.3	0.5	0.2	0.3
Vigilance	1.3	0.7	2.1^*^	0.9	1.7	0.8	1.6	0.9
DoC-NP score								
Total	4.8	2.3	6.3^*^	1.7	5.8	2.1	5.1	2.2
EEG–continuity	1.5	0.8	1.8	0.7	1.6	0.7	1.6	0.7
EEG–AFR	1.6	1.0	2.3^*^	1.1	2.0	1.0	1.8	1.1
SEPs–N20	0.8	0.7	1.2^*^	0.5	1.0	0.7	0.9	0.7
ERPs–P300	0.9	0.7	1.1^*^	0.6	1.1	0.6	0.9	0.7

VS/UWS, vegetative state/unresponsive wakefulness syndrome; MCS, minimal conscious state; SD, standard deviation; CRS-R, coma recovery scale-revised; EEG, electroencelography; SEPs, somatosensory evoked potentials; ERPs, evoked-related potentials. ^*^p < 0.05.

### 3.1 Prognostic ability of the DoC-NP Score—Full clinical recovery of consciousness outcome

**Figure 2:**
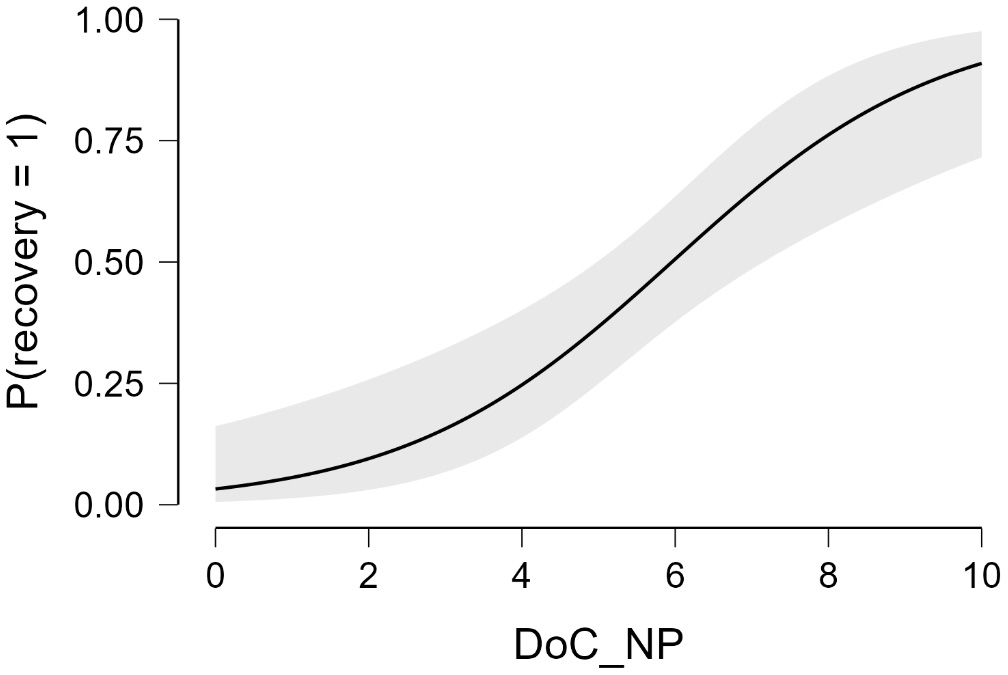
Probability of recovery as a function of DoC-NP score estimated from logistic regression. The shaded area is the 95% confidence interval of the curve.

The prognostic ability of DoC-NP Score to predict full recovery of clinical consciousness with eMCS was tested with a logistic regression model. Figure 2 shows the probability of clinical consciousness recovery based on the DoC-NP Score.

The *OR* of clinical recovery was 1.768 (95% CI 1.317–2.373), and the likelihood of recovery increased by approximately 76.8% for each one-unit increase in the DoC-NP Score. Even after adjusting for age and status at baseline (MCS vs. VS/UWS), the *OR* of recovery remained significant (aOR = 1.701, 95% CI 1.205–2.426).

Moreover, a logistic regression analysis was carried out in order to stratify by UWS/MCS state. The prediction was significant in VS/UWS patients (*OR* = 1.658, 95% CI 1.103–2.491, *p* < 0.05), but failed to reach significance in MCS patients (*OR* = 1.764, 95% CI 0.936–3.326, *p* = 0.07).

**Figure 3:**
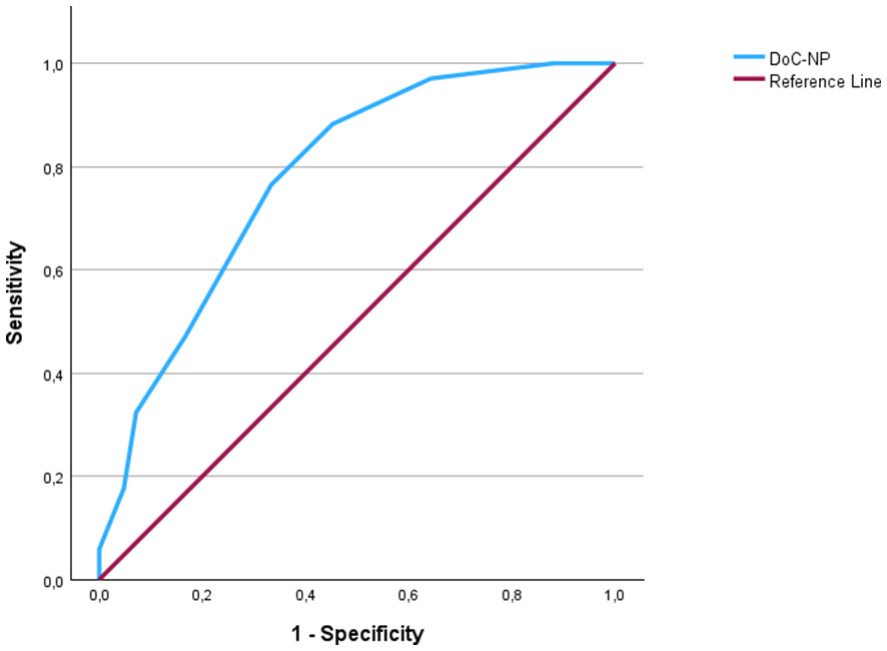
ROC curve showing the performance of the DoC-NP score.

Using ROC analysis, we found that the performance of the DoC-NP Score as a predictor of recovery was good, with an AUC of 0.780 (95% CI 0.678–0.883) as shown in Figure 3. The optimal cut-off for the score was 6, with a sensitivity of 76.5% and a specificity of 66.7%. The overall accuracy of the model was 71.1%.

The *OR* of recovery for CRS-R was 1.553 (95% CI 1.239–1.947, *p* < 0.01) and that for DoC-NP Score was 1.526 (95% CI 1.088–2.140, *p* < 0.05), using a stepwise logistic regression model. The Nagelkerke R^2^ increased from 50.1% to 57.3%, and the percentage of cases correctly classified from 81.6% to 85.5%.

### 3.2 Correlation between DoC-NP Score and its sub-items

**Figure 4:**
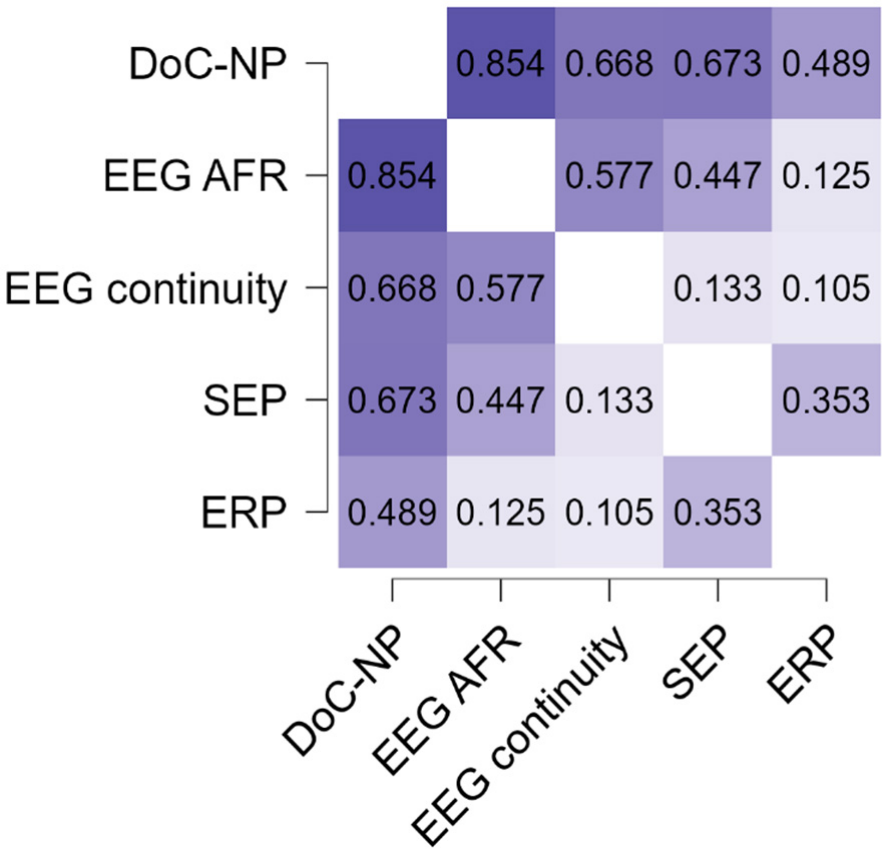
Spearman's rho heatmap showing the correlations between the DoC-NP Score and its components. Correlations were interpreted as weak (< 0.30), moderate (0.30–0.50), or strong (>0.50). CRS-R, coma recovery scale-revised; DoC-NP score, Disorder of Consciousness-neurophysiological score; EEG, electroencelography; AFR, amplitude-frequency-reactivity; SEPs, somatosensory evoked potentials; ERPs, evoked-related potentials.

The Spearman's rho heatmap (Figure 4) shows, with color intensity corresponding to the strength of the correlation, that the DoC-NP Score strongly correlates (>0.50) with EEG AFR (*r* = 0.854) and EEG continuity (*r* = 0.668) and SEPs (*r* = 0.673); moderate (0.3> *r* >0.5) with ERPs (*r* = 0.489).

### 3.3 Correlation between DoC-NP Score and changes in the CRS-R total score and its sub-items (Δ_CR−R_)

Table 3 shows that the DoC-NP Score correlates with the change in CRS-R total score and all its components except for vigilance (*p* = 0.148). More in deep, DoC-NP Score moderate correlates with the Δ_CRS − R_ (*r* = 0.434, *p* < 0.001), auditory function (*r* = 0.445, *p* < 0.001), motor function (*r* = 0.435, *p* < 0.001), motor oral/verbal function (*r* = 0.334, *p* = 0.003), communication (*r* = 0.315, *p* = 0.006); a poor correlation, instead, has been showed with visual function (*r* = 0.270, *p* = 0.018). As shown in Table 3, the DoC-NP Score has a better correlation with the changes in the CRS-R and its subscales from T0 to T1, compared to each neurophysiological sub-items of the DoC-NP Score.

**Table 3:** Spearman's rho correlations of the DoC-NP Score and its neurophysiological sub-items with changes in CRS-R total (ΔCRS-R) and its subscales from admission to discharge.

Correlations	ΔCRS-R total	ΔAuditory	ΔVisual	ΔMotor	ΔOromotor verbal	ΔCommunication	ΔVigilance
DoC-NP Score	0.434^*^	**0.445** ^*^	**0.270** ^*^	**0.435** ^*^	**0.334** ^*^	**0.315** ^*^	0.168
EEG continuity	**0.306** ^*^	**0.262** ^*^	**0.269** ^*^	**0.225** ^*^	0.185	**0.272** ^*^	0.170
EEG AFR	**0.416** ^*^	**0.386** ^*^	**0.345** ^*^	**0.359** ^*^	**0.385** ^*^	**0.295** ^*^	0.110
SEPs	0.182	0.225	0.012	**0.275** ^*^	0.134	0.109	0.011
ERPs	**0.299** ^*^	**0.361** ^*^	**0.070**	**0.354** ^*^	0.145	0.210	**0.275** ^*^

Correlations were interpreted as weak (< 0.30), moderate (0.30–0.50), or strong (>0.50). Bold values: ^*^p < 0.05, statistical significance.

### 3.4 Prognostic ability of the DoC-NP Score—Functional outcomes

First, we investigated the prognostic ability of the DoC-NP Score using it as a predictor of the LCF score at discharge (T1), in a linear regression model, adjusted for the LCF score at baseline (T0). We found that DoC-NP Score predicted this outcome above and beyond the baseline value of the LCF. The regression coefficients were positive, meaning that higher neurophysiological scores were associated with higher LCF. The model explained 31.8% of the variance of LCF at T1, with 23.8% explained by LCF, and 7.9% by the DoC-NP Score, as shown in Table 4.

**Table 4:** Stepwise linear regression model of LCF at discharge (T1) on the DoC-NP score.

Model		Unstandardized coefficients		Standardized coefficients	*t*	Sig.
		*B*	SE(*B*)	Beta		
1	(Constant)	0.643	0.747		0.861	0.392
	LCF at baseline	1.476	0.307	0.488	4.814	< 0.001
2	(Constant)	−0.083	0.761		−0.109	0.914
	LCF at baseline	1.249	0.304	0.413	4.103	< 0.001
	DoC-NP score	0.199	0.071	0.281	2.793	**0.007** ^*^

Results are reported as unstandardized and standardized regression coefficients. LCF, Levels of cognitive functions; SE, standard error. Bold values: ^*^p < 0.05, statistical significance.

Second, we analyzed the prognostic ability of the DoC-NP Score using it as a predictor of DRS score at T1 in a linear regression model, adjusted for the DRS score at baseline. The regression coefficient was negative, meaning that higher DoC-NP Scores were associated with lower disability. The model explained 30.8% of the variance of DRS at discharge, with 25.8% of DRS, and 5% by the DoC-NP Score, as shown in Table 5.

**Table 5:** Stepwise linear regression model of DRS at discharge (T1) on the DoC-NP score.

Model		Unstandardized coefficients		Standardized coefficients	*t*	Sig.
		B	Std. error	Beta		
1	(Constant)	−5.903	4.734		−1.247	0.216
	DRS at baseline	1.013	0.200	0.508	5.076	< 0.001
2	(Constant)	2.666	6.174		0.432	0.667
	DRS at baseline	0.795	0.221	0.399	3.598	< 0.001
	DoC-NP score	−0.541	0.258	−0.233	−2.098	**0.039** ^*^

Results are reported as unstandardized and standardized regression coefficients. DRS, Disease Recovery Scale; SE, standard error. Bold values: ^*^p < 0.05, statistical significance.

## 4 Discussion

This retrospective study evaluated the clinical utility of a novel multiparametric neurophysiological score—the DoC-NP Score—developed to support prognostic assessment of recovery of a clinically observable state of consciousness during rehabilitation in patients with pDoC. By integrating complementary neurophysiological markers into a single composite index, the DoC-NP Score offers a structured and clinically applicable framework for summarizing residual brain function. Traditionally, prognostic outcomes in DoC research have relied on categorical changes in diagnostic status based on CRS-R classifications (43). However, this approach may create interpretative ambiguity, particularly when changes are subtle yet clinically meaningful (36, 44). In this context, eMCS represents a more robust and clinically relevant endpoint for defining recovery of consciousness, as increasingly recognized in recent literature (40). eMCS is operationally defined by the recovery of functional use of objects or functional communication, reflecting a clear transition toward purposeful interaction with the environment (6). Importantly, the outcome examined in this study should be understood as recovery of a full clinically observable conscious brain state, operationally defined by eMCS criteria. This concept differs from the broader notion of full human consciousness, which refers to a complex mental capacity encompassing higher-order cognitive abilities, intentionality, and self-awareness (9). In the context of DoC, clinical assessment necessarily relies on behavioral indicators of conscious interaction with the environment rather than on the complete restoration of the spectrum of human mental capacities.

Previous studies have demonstrated the prognostic relevance of baseline CRS-R scores for short-term improvement of consciousness (45). More recently, clinical guidelines and empirical evidence have highlighted the added value of multimodal assessment strategies, recommending that behavioral evaluations be complemented by at least one instrumental measure, particularly in patients diagnosed as UWS or MCS after sABI (1, 13, 23, 46, 47).

Bodien et al. indicate that the most accurate diagnostic frameworks in DoC rely on multimodal approaches combining behavioral examination, electrophysiological measures, and advanced neuroimaging, as well as paradigms capable of detecting covert awareness, also known as cognitive motor dissociation (CMD) (22), functional Locked-In Syndrome (fLIS) (48) or Non-Behavioral MCS (MCS^*^) (24). Moreover, the availability of such comprehensive assessments may vary across post-ICU and neurorehabilitation settings, as they are largely dependent on local infrastructures, resources and specialized expertise. Furthermore, prognostic approaches in pDoC remain imperfect and continue to evolve for a better diagnosis and prognostication (49).

Emerging evidence suggests that recovery trajectories differ substantially in patients with early evidence of CMD, with those exhibiting covert consciousness showing a higher likelihood of long-term recovery following rehabilitation compared to patients without CMD. These findings support the role of advanced neurophysiological and neuroimaging markers in identifying patients who may benefit from more intensive and targeted neurorehabilitation strategies (50).

In line with this perspective, recent frameworks (such as the AVC(M) score) integrate clinical examination, structural imaging, and both resting-state and task-based assessments of responsiveness, incorporating dimensions of arousal, volitional motor output, and cognitive content, including CMD. Within this multimodal paradigm, clinically applicable tools that provide standardized and complementary prognostic information—such as neurophysiological composite scores—may support prognostic stratification in routine rehabilitation settings by contributing structured bedside data alongside clinical and neuroimaging assessments (37).

For this reason, the present study intentionally focused on developing a prognostic score based on neurophysiological parameters, including EEG, SEPs, and ERPs, with the aim of providing a structured and clinically applicable tool that may complement the aforementioned AVC(M) framework in routine practice. Previous neurophysiological scoring approaches have been proposed in the literature, such as the AFR score described by Bagnato et al., primarily based on EEG background organization (25). Building on this concept, we aimed to extend the neurophysiological assessment by incorporating additional electrophysiological markers relevant for prognostication in DoC. Although several of these markers may show limited prognostic performance when considered individually, their integration within a composite score may capture complementary aspects of thalamocortical and cortico-cortical network integrity underlying recovery of consciousness (12, 19).

EEG is among the most commonly used neurophysiological modalities in clinical neuroprognostication, although its availability and interpretation in neurorehabilitation settings may still depend on technical expertise and resources (51). Besides that, EEG recordings obtained early after admission to ICUs or IRUs, and interpreted according to standardized ACNS terminology, have been shown to provide meaningful prognostic information (52). In particular, background activity and continuity patterns—such as those captured by Hockaday, Synek or Young and AFR classifications—have been associated with subsequent responsiveness and cognitive recovery (53, 54). Similarly, EEG reactivity to external stimulation has been linked to a higher likelihood of short-term consciousness improvement (44). In contrast, sporadic epileptiform discharges (SEDs)—abnormal, non-rhythmic transient electrical waves distinct from background activity and reflecting neuronal hyperexcitability—have not been consistently associated with the level of responsiveness or clinical prognosis in patients with pDoC, and were therefore not included in the DoC-NP Score (55).

In parallel, upper limbs SEPs provide essential information on the integrity of thalamocortical pathways, which play a central role in sensory integration and cortical processing. In VS/UWS, these pathways may be disrupted by neuronal loss after hypoxic–ischemic injury or by axonal damage following TBI (30). Thalamocortical dysfunction is considered a core mechanism underlying disorders of consciousness, and recovery is thought to depend on plastic reorganization within these connections (29). In this context, the bilateral absence of the cortical N20 component is widely recognized as a robust marker of poor prognosis, being associated with an almost null probability of recovery after hypoxic injury and a very low recovery rate after TBI (28, 42).

Conversely, the presence of early-EPs indicates preserved sensory pathway integrity but does not, by itself, guarantee a favorable outcome. For this reason, long-latency ERPs have been introduced into prognostic assessment, as they reflect higher-order cortical processing (51). Auditory paradigms can elicit ERPs even without explicit task engagement, providing insight into multiple stages of sensory and cognitive processing. The N100 component reflects early auditory cortical activation, whereas the P300 is associated with stimulus evaluation, working memory updating, and the allocation of attentional resources, making it a plausible marker of higher cognitive function and potential conscious awareness (56). Several studies have shown that the presence of a P300 is associated with a greater likelihood of recovery from coma or VS/UWS; however, its absence does not reliably predict poor outcome, partly due to limited sensitivity and substantial inter- and intra-individual variability (38, 57, 58).

In our cohort of 76 patients with pDoC, nearly half (46%) achieved eMCS by discharge, with no significant association between recovery and injury etiology. The lack of association between etiology and recovery of full clinical consciousness in our cohort should be interpreted with caution. Although TBI is often associated with a more favorable prognosis compared to non-traumatic etiologies, this dichotomous classification may oversimplify the heterogeneity within the non-TBI group (4). In particular, vascular brain injuries may show recovery trajectories comparable to TBI, whereas anoxic injuries are typically associated with poorer outcomes (4, 59). In our study, the absence of a significant effect may also reflect a selection bias related to the rehabilitation setting. Specifically, anoxic patients were pre-selected prior to admission, resulting in a “filtered” population that is not fully representative of the broader pDoC population described in acute care settings (60).

The mean DoC-NP Score at admission was six and showed a positive correlation with age, but not with etiology. This finding likely reflects the etiological distribution within our sample: younger patients were more frequently affected by TBI or post-anoxic cardiac arrest—conditions associated with more severe neurophysiological impairment—whereas older patients presented with ischemic or hemorrhagic stroke, which tended to yield higher CRS-R scores at admission. As expected, patients diagnosed with MCS at baseline exhibited significantly higher DoC-NP Scores and better behavioral performance compared with those in VS/UWS. The positive correlation between DoC-NP Score and age appears counterintuitive but can be explained by the etiological distribution of the cohort. Younger patients were more frequently affected by diffuse injuries, such as traumatic axonal injury or post-anoxic damage, which disproportionately disrupt thalamocortical and cortico-cortical connectivity and are associated with lower neurophysiological scores. In contrast, older patients more often presented with focal vascular lesions, which may spare key hubs of the consciousness network, resulting in relatively preserved neurophysiological markers despite advanced age.

The finding that the DoC-NP Score showed more predictive performance in VS/UWS compared to MCS patients may appear counter-intuitive, given that MCS is clinically closer to recovery. However, this observation is consistent with previous studies, including Steppacher et al. and recent meta-analytic evidence by Pavlov et al., suggesting that outcome prediction in MCS is inherently more challenging (4, 61). From a network perspective, VS/UWS may reflect a more globally disrupted state of thalamocortical and cortico-cortical connectivity, in which the presence or absence of residual functional integrity can be more clearly captured by multimodal assessment (62). In contrast, MCS is characterized by partial and often unstable reorganization of large-scale brain networks, resulting in more variable patterns of connectivity and less predictable recovery trajectories, thereby reducing the discriminative power of prognostic models (63, 64). Furthermore, these findings may be interpreted within the framework of biological endotypes rather than clinical phenotypes, supporting a precision medicine approach in which patients are stratified based on underlying pathophysiological mechanisms rather than behavioral diagnosis alone (65). Such heterogeneity may partly explain why prognostic stratification in MCS remains particularly challenging despite its apparent clinical proximity to recovery. The higher DoC-NP Scores observed in patients diagnosed with MCS compared with VS/UWS further support the construct validity of the score, as MCS is associated with partial preservation of distributed frontoparietal and thalamocortical networks underlying intentional behavior. Consistently, the strong association between increasing DoC-NP Scores and the probability of achieving eMCS suggests that the score may reflect a critical threshold of network integration beyond which functional communication or object use becomes possible. The identified cut-off of 6 may therefore reflect a minimal level of neurophysiological integrity required for the re-emergence of clinical conscious awareness, irrespective of injury etiology.

Correlation analyses indicated that EEG background organization (AFR score and continuity) and N20 SEPs accounted for most of the variance in the DoC-NP Score, underscoring the central role of preserved thalamocortical and large-scale cortical dynamics in consciousness recovery. In contrast, ERPs showed a moderate contribution. Moreover, additional analysis showed that the DoC-NP Score was significantly associated with behavioral recovery over time, correlating with changes in total CRS-R score and with all CRS-R subdomains except for vigilance. Moderate correlations were observed with the gain of the CRS-R total score at discharge, auditory, motor, motor–oral/verbal, and communication functions, whereas only a weak association emerged with visual function. This pattern suggests that the neurophysiological processes captured by the DoC-NP Score primarily reflect the integrity of distributed thalamocortical and frontoparietal networks supporting purposeful behavior and communication, rather than arousal regulation or isolated sensory responsiveness.

These findings support the conceptual premise that combining multiple complementary neurophysiological markers may enhance prognostic accuracy when integrated with other clinical and instrumental measures. Importantly, these results also underscore the clinical complexity of distinguishing between recovery of consciousness and broader functional outcomes, particularly when counseling families. While recovery of consciousness implies restoration of communication, reductions in disability and gains in autonomy often represent the most meaningful outcomes for patients and caregivers when planning post-IRU care pathways (66).

To further elucidate the relationship between neurophysiology and behavior, we examined correlations between CRS-R subscales and both the composite score and individual neurophysiological markers. Moderate associations were observed between the DoC-NP Score and the motor and communication subscales—the only CRS-R domains used to establish a diagnosis of eMCS—supporting the clinical relevance of the score. In contrast, although visual function also correlated moderately with the composite score, this association did not contribute to the clinical identification of eMCS.

Finally, the DoC-NP Score demonstrated meaningful associations with functional outcomes at discharge. It explained a greater proportion of variance in LCF scores than in DRS scores, suggesting greater sensitivity to cognitive recovery than to global disability. Overall, these findings indicate that the DoC-NP Score performs well across multiple clinically relevant endpoints, including recovery of consciousness, cognitive improvement, and functional outcome.

Overall, these findings suggest that the DoC-NP Score may represents a clinically useful complementary tool for summarizing neurophysiological information and may contribute to improved prognostic stratification when used within a multimodal assessment framework stratification in patients with pDoC undergoing rehabilitation, although its practical implementation and resource implications may vary across clinical settings.

### 4.1 Study limitations

The retrospective design and relatively limited sample size may introduce selection bias and limit generalizability.

A further limitation concerns the timing and execution of the neurophysiological battery that was performed over multiple days rather than at a single time point. This may have introduced temporal variability in patients' clinical status, as subtle changes in the level of consciousness may have occurred between baseline behavioral assessment and completion of electrophysiological testing. Although daily CRS-R evaluations did not reveal clear evidence of such changes, the possibility of interim clinical improvement cannot be entirely excluded.

Moreover, the use of discharge (T1) as the primary outcome timepoint may introduce temporal bias in outcome determination, as the exact timing of eMCS could not be precisely identified, potentially leading to underestimation or misclassification of recovery trajectories.

Fluctuations in arousal—either spontaneous or medication-related—may have influenced neurophysiological measures, particularly ERPs such as the P300, which require adequate attentional engagement, thereby contributing to variability in DoC-NP Scores.

Importantly, the incremental prognostic value of the DoC-NP Score beyond the CRS-R was modest, with only a limited increase in explained variance and classification accuracy. This finding suggests that while neurophysiological measures may provide complementary information, they are unlikely to replace behavioral assessment and should be interpreted within a multimodal framework. It also highlights the need for continued refinement of neurophysiological predictors and for the development of more integrative models capable of achieving clinically meaningful improvements in prognostic accuracy.

Future studies should aim to standardize assessment timing and, when feasible, perform evaluations at least 12 h after administration of centrally acting medications. In addition, the absence of other neurophysiological measures—such as mismatch negativity (MMN), visual evoked potentials (VEPs), and brainstem auditory evoked potentials (BAEPs)—represents a further limitation. MMN, in particular, is a pre-attentive marker of auditory discrimination and has been proposed as an early positive predictor of recovery, whereas the prognostic value of the P300 remains controversial due to limited sensitivity and methodological heterogeneity. Furthermore, the low test–retest reliability of ERPs and marked intra-individual fluctuations in vigilance suggest that negative ERP findings should be interpreted with caution and ideally confirmed through longitudinal assessments (19). Another limitation of standard ERP paradigms is their reliance on relatively neutral auditory stimuli, which may fail to engage sufficient cortical resources in patients with impaired vigilance. More salient stimuli, such as the subject's own name, have been shown to increase the probability of eliciting P300 responses and may improve the sensitivity of ERP-based prognostication. The lack of such paradigms in the present study may therefore have led to an underestimation of preserved cognitive processing in some patients. Accordingly, a single negative ERP assessment should not be considered conclusive evidence of impaired consciousness, underscoring the need for repeated or longitudinal evaluations.

Given that no single neurophysiological measure currently represents a gold standard for prognostication in pDoC, future research should focus on multidimensional approaches integrating multiple neurophysiological, behavioral and neuroimaging markers to improve prognostic accuracy. Taken together, these considerations reinforce the need for multidimensional prognostic frameworks that integrate clinical behavioral, neurophysiological, and neuroimaging data, rather than relying on any single marker, to improve prognostic accuracy in patients with pDoC

### 4.2 Future directions

The present retrospective observational study should be regarded as exploratory and hypothesis-generating. Future research should prioritize the inclusion of larger cohorts to improve the representativeness of the sample and enhance the generalizability of the findings.

Equally important will be the standardization of the timing of neurophysiological recordings and clinical assessments, in order to limit the confounding effects of arousal fluctuations and centrally acting medications. In this context, longitudinal study designs with predefined assessment time points would allow a more accurate characterization of the temporal evolution, stability, and prognostic value of neurophysiological and behavioral markers across the rehabilitation trajectory.

Subsequent investigations should also expand the neurophysiological battery by incorporating additional measures, such as mismatch negativity (MMN), visual evoked potentials (VEPs), and brainstem auditory evoked potentials (BAEPs). These techniques may provide complementary insights into sensory pathway integrity and higher-order cognitive processing. Exploring their associations with CRS-R subscales may further refine the diagnostic and prognostic performance of the DoC-NP Score.

In this context, the DoC-NP Score was conceived as a potentially applicable neurophysiological tool to support prognostic stratification in patients with pDoC admitted to neurorehabilitation units, while future studies will be needed to determine whether integration with clinical variables and neuroimaging findings within multimodal frameworks can further improve its predictive performance.

Future studies should integrate sleep-related neurophysiological assessments, which were not available in the present dataset. In particular, full polysomnographic recordings would enable the evaluation of EEG abnormalities during sleep and the detection of NREM sleep graphoelements, such as sleep spindles and K-complexes (32, 67). The presence or re-emergence of these markers has been increasingly recognized as indicative of preserved thalamocortical function and has been associated with earlier recovery of consciousness in patients with prolonged disorders of consciousness, underscoring their potential prognostic relevance (68, 69).

Future prospective studies should also evaluate the real-world feasibility, implementation burden, and cost-effectiveness of neurophysiological prognostic tools across different healthcare systems.

Moreover, external validation in independent, prospectively collected cohorts will be essential to confirm the reproducibility, and clinical applicability of the proposed score.

Finally, future prospective longitudinal studies should incorporate standardized and temporally aligned assessments—capturing the earliest clinical evidence of eMCS and extending follow-up beyond hospital discharge, including structured remote evaluations—and perform neurophysiological testing in close temporal proximity to neuroimaging and behavioral assessments, ideally with CRS-R evaluation immediately preceding testing, in order to more accurately characterize recovery trajectories and minimize temporal bias in outcome determination; moreover, such approaches should be extended across different clinical settings, including ICU, to capture neurophysiological data during the acute phase and enable a longitudinal characterization of recovery trajectories across acute, post-acute, and chronic stages of disorders of consciousness.

## 5 Conclusions

This retrospective observational study assessed the potential clinical utility of the DoC-NP Score as a feasible multiparametric neurophysiological tool that may complement existing prognostic approaches for evaluating prognostication in patients with pDoC during the post-acute rehabilitation phase. By integrating standard bedside EEG features with somatosensory and cognitive evoked potentials, the DoC-NP Score showed a modest capacity to predict recovery of full consciousness (eMCS) and functional outcomes at discharge, the strongest associations observed for CRS-R behavioral domains.

Although the limited sample size and retrospective design warrant caution and external validation is required, these findings suggest that the integration of complementary neurophysiological markers into a single composite score may improve prognostic stratification beyond isolated measures.

Importantly, the DoC-NP Score should be interpreted as one component of a multimodal prognostic approach, in which neurophysiological, behavioral, and neuroimaging markers are integrated to improve diagnostic accuracy and outcome prediction, in line with current recommendations.

## Data Availability

The raw data supporting the conclusions of this article will be made available by the authors, without undue reservation.
